# Pharmacological Inhibition of Inward Rectifier Potassium Channels Induces Lethality in Larval *Aedes aegypti*

**DOI:** 10.3390/insects9040163

**Published:** 2018-11-15

**Authors:** Renata Rusconi Trigueros, Corey R. Hopkins, Jerod S. Denton, Peter M. Piermarini

**Affiliations:** 1 Department of Entomology, The Ohio State University, Ohio Agricultural Research and Development Center, Wooster, OH 44691, USA; renata.rusconi21@gmail.com; 2 Department of Pharmaceutical Sciences, University of Nebraska Medical Center, Omaha, NE 68198, USA; corey.hopkins@unmc.edu; 3 Departments of Anesthesiology and Pharmacology, Vanderbilt University Medical Center, Nashville, TN 37232, USA; jerod.s.denton@vumc.org

**Keywords:** mosquito, Kir channel, barium, VU041, small molecule, insecticide

## Abstract

The inward rectifier potassium (Kir) channels play key roles in the physiology of mosquitoes and other insects. Our group, among others, previously demonstrated that small molecule inhibitors of Kir channels are promising lead molecules for developing new insecticides to control adult female mosquitoes. However, the potential use of Kir channel inhibitors as larvicidal agents is unknown. Here we tested the hypothesis that pharmacological inhibition of Kir channels in the larvae of *Aedes aegypti*, the vector of several medically important arboviruses, induces lethality. We demonstrated that adding barium, a non-specific blocker of Kir channels, or VU041, a specific small-molecule inhibitor of mosquito Kir1 channels, to the rearing water (deionized H_2_O) of first instar larvae killed them within 48 h. We further showed that the toxic efficacy of VU041 within 24 h was significantly enhanced by increasing the osmolality of the rearing water to 100 mOsm/kg H_2_O with NaCl, KCl or mannitol; KCl provided the strongest enhancement compared to NaCl and mannitol. These data suggest: (1) the important role of Kir channels in the acclimation of larvae to elevated ambient osmolality and KCl concentrations; and (2) the disruption of osmoregulation as a potential mechanism of the toxic action of VU041. The present study provides the first evidence that inhibition of Kir channels is lethal to larval mosquitoes and broadens the potential applications of our existing arsenal of small molecule inhibitors of Kir channels, which have previously only been considered for developing adulticides.

## 1. Introduction

Mosquitoes are vectors of numerous pathogens that debilitate the health and well-being of humans and vertebrate animals. The yellow fever mosquito *Aedes aegypti* is the principal vector of the chikungunya, dengue, yellow fever, and Zika viruses. These arboviruses collectively infect hundreds of millions of people around the globe each year, resulting in hundreds of thousands of hospitalizations and tens of thousands of deaths [1]. A common strategy to prevent the transmission of these pathogens is to control mosquito populations, which often involves the use of chemical insecticides. However, the overuse of insecticides with limited modes of neurotoxic action (e.g., pyrethroid modulation of voltage-gated Na^+^-channels and carbamate inhibition of acetylcholinesterase) has led to target site and metabolic resistance, thereby reducing the efficacy of mosquito control [2,3]. Thus, the discovery and development of insecticides with novel mechanisms of toxic action are needed to supplement our toolbox for vector control.

Since 2011, our group has pursued the discovery of small molecule inhibitors of mosquito inward rectifier potassium (Kir) channels in the context of developing novel insecticides for controlling adult female mosquitoes [4,5]. Kir channels are recognized to play diverse physiological roles in insects [6,7,8,9,10,11,12,13,14,15,16,17,18,19,20,21,22,23]. In adult female mosquitoes, Kir channels are especially important in: (1) transepithelial K^+^ and fluid secretion in Malpighian (renal) tubules; (2) diuresis; (3) hemolymph volume and K^+^ homeostasis; (4) blood meal processing; and (5) egg production [7,8,9,10,11,13,19,24,25]. Thus, Kir channel inhibition is expected to kill mosquitoes or disrupt their life cycle via novel mechanisms of action from existing neurotoxic insecticides.

To date, we have discovered hundreds of small molecule inhibitors of mosquito Kir1 channels and have focused on testing the insecticidal efficacy of four molecules with unique chemical scaffolds: VU573, VU590, VU625 and VU041. All four are toxic to adult female mosquitoes when injected into the hemolymph or applied topically to the cuticle [8,13,24,25]. Notably, one of these small molecules (VU041) is equally efficacious against pyrethroid-susceptible and pyrethroid-resistant strains of *Ae. aegypti* and *Anopheles gambiae* and does not show apparent topical toxicity against a representative pollinator, the honey bee *Apis mellifera* [13]. VU041 has recently been shown to kill and reduce fecundity of *Anopheles quadrimaculatus* in semi-field conditions [26]. Thus, VU041 offers a promising chemical scaffold for mosquitocide development.

Despite the promise of Kir channel inhibitors as adulticides, little is known about their potential use as larvicides. Using qPCR, we have shown that the expression of mRNAs encoding various Kir channel subunits was of similar or greater abundance in larval *Ae. aegypti* relative to adult females [21]. Moreover, the mRNA expression of one or more Kir subunits was typically enriched in key osmoregulatory tissues of *Ae. aegypti* larvae, such as the midgut, Malpighian tubules, and anal papillae [21]. In some cases, Kir subunit mRNAs were differentially expressed in fourth instar larvae when reared in water with an elevated KCl concentration [21]. Thus, molecular data suggest Kir channels play an important role in larval osmotic and ionic homeostasis. A recent study demonstrated that flonicamid, a small molecule inhibitor of hemipteran Kir1 channels [19], was nominally toxic to third instar larvae of *Ae. aegypti* and *An. gambiae*, albeit the weak cuticular penetration of this compound likely limited its efficacy [27].

The goal of the present study was to test the hypothesis that pharmacological inhibition of Kir channels in larval mosquitoes using barium or VU041 would disrupt osmotic and/or ionic homeostasis, leading to death. We found that exposing mosquito larvae to barium or VU041 killed them within 48 h. Moreover, we demonstrated that the efficacy of VU041 as a larvicide was enhanced by elevating concentrations of ambient NaCl, KCl, or mannitol, suggesting a role of Kir channels in larval osmotic and ionic homeostasis. Our study is the first to demonstrate that Kir channel inhibition is a mode of action for killing larval mosquitoes. 

## 2. Materials and Methods

### 2.1. Mosquito Colony

The *Ae. aegypti* colony (Liverpool strain) used for the present study was derived from eggs provided by the MR4 as part of the BEI Resources Repository, NIAID, NIH (LVP-IB12, MRA-735, deposited by M.Q. Benedict). The eggs were hatched in deionized H_2_O (dH_2_O) and raised to adulthood as previously described [28]. To produce additional eggs, adult females were fed defibrinated rabbit blood (purchased commercially from Hemostat Laboratories, Dixon, CA, USA) via a membrane feeder (Hemotek, Blackburn, UK).

### 2.2. Chemicals

The synthesis of the small molecules VU041 and VU937 was described previously [13]. 10 mM stock solutions of the small molecules were prepared in 100% dimethyl sulfoxide (DMSO). Barium chloride (BaCl_2_), sodium chloride (NaCl), potassium chloride (KCl), and mannitol were all purchased from Fisher Thermo Scientific (Waltham, MA, USA). Stock solutions of BaCl_2_ were prepared in dH_2_O at various concentrations.

### 2.3. Larval Toxicity Bioassays

The larval toxicity assays were performed following an established assay [28,29,30]. Six larvae were transferred to the wells of a 24-well plate (Falcon Multiwell plate, Becton Dickinson Labware, Franklin Lakes, NJ, USA) containing 985 μL of dH_2_O, 50 mM NaCl, 50 mM KCl, or 100 mM mannitol. Each well also contained 5 μL of a food solution consisting of 13 mg/mL of finely ground Tetramin flakes (Blacksburg, VA, USA) suspended in dH_2_O. To each well, 10 μL of BaCl_2_, VU041, or VU937 were added. Control wells for BaCl_2_ treatment received 10 μL of dH_2_O, whereas control wells for small molecules received 10 μL of 100% DMSO (resulting in a final concentration of 1% DMSO). The plates were placed in a rearing chamber (28 °C, 80% relative humidity, 12 h:12 h light:dark) and assessed for survival at 24 h and 48 h. Larvae were considered dead if they did not move after a gentle prod with a fine insect pin or pipette tip. In experiments testing the effects of 50 mM NaCl, 50 mM KCl, or 100 mM mannitol, the osmolality of the rearing water was confirmed to be 100 ± 5 mOsm/kg H_2_O using a vapor pressure osmometer (Wescor, Logan, UT, USA).

### 2.4. Statistics

GraphPad Prism 6 (La Jolla, CA, USA) was used for all statistical analyses. The specific tests are described in the figure legends.

## 3. Results

**Figure 1:**
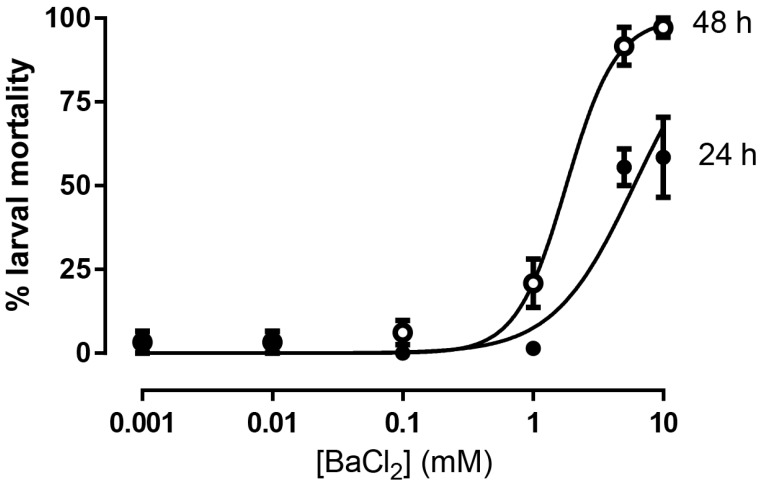
Concentration-toxicity relationship of BaCl_2_ in first instar *Aedes aegypti* at 24 h and 48 h after addition to the rearing water (dH_2_O). Values are means ± standard error of the mean (SEM) based on six replicates of six larvae per concentration. Control mortality in dH_2_O without BaCl_2_ was 0% within 24 h and 2.8 ± 2.8% within 48 h (*n* = 6 replicates of 6 larvae). The 48 h LC_50_ was determined with a ‘log(agonist) vs. normalized response-Variable slope’ curve fit in GraphPad Prism 6.

To establish proof of concept that inhibition of Kir channels was potentially toxic to larvae, we tested the effects of adding barium, a non-selective blocker of Kir channels, to the rearing water (dH_2_O). As shown in Figure 1, BaCl_2_ treatment caused concentration-dependent mortality within 24 h, but only reached a maximum efficacy of ~60% at 10 mM, the highest concentration tested. By 48 h, barium-induced mortality approached 100% with a median lethal concentration (LC_50_) of 1.8 mM (95% C.I. = 1.35–2.385 mM) (Figure 1).

**Figure 2:**
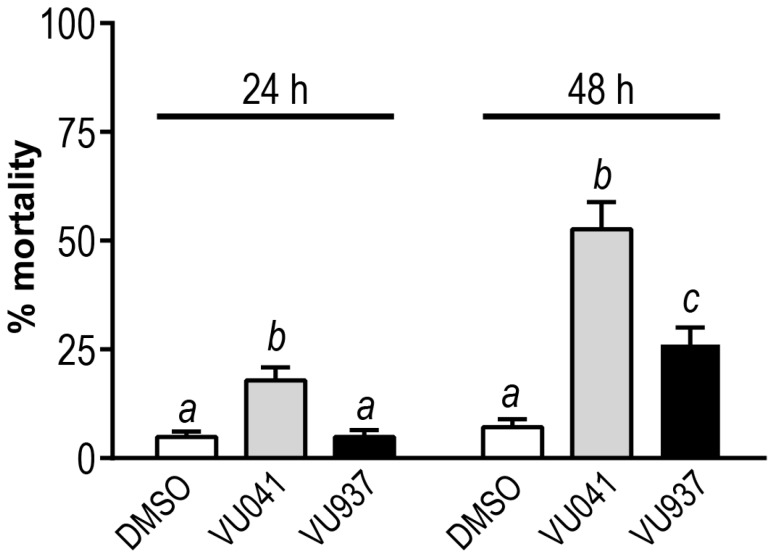
Toxicity of VU041 and VU937 in first instar *Ae. aegypti* at 24 h and 48 h after addition to the rearing water (dH_2_O). The concentrations of the VU small molecules and DMSO control were 100 µM and 1%, respectively. Values are means ± SEM based on 42 replicates of six larvae per treatment. Lower-case letters indicate statistical categorization of the means within each time point as determined by two-way repeated measures ANOVA with a Boneferroni’s multiple comparisons test (*p* < 0.05).

We next tested whether VU041, a selective small molecule inhibitor of mosquito Kir1 channels, would induce toxicity in larvae. In parallel, we tested an analog of VU041 (VU937) that is a less potent in vitro mosquito Kir1 inhibitor than VU041 and is less toxic to adult female mosquitoes than VU041 [13,20]. We used a concentration of 100 μM for VU041 and VU937, which was at VU041’s solubility limit in water. As shown in Figure 2, the VU041 treatment resulted in limited, but significant, mortality within 24 h compared to the DMSO control and the VU937 treatment. The toxicity of VU041 at 24 h was ~3.6 times greater than that of DMSO and VU937. By 48 h, VU041 elicited over 50% mortality, which was significantly greater than the mortalities induced by DMSO and VU937 (Figure 2). The toxicity of VU041 at 48 h was ~7.3 times greater than DMSO and 2 times greater than VU937. The toxicity of VU937 at 48 h was significantly, 3.6 times, greater than the DMSO control (Figure 2).

**Figure 3:**
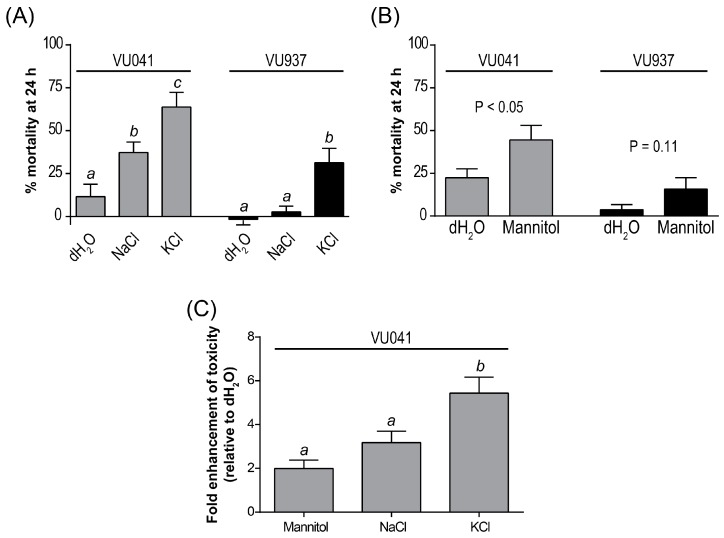
(**A**) Effects of adding 50 mM NaCl or KCl to the rearing water on the 24 h toxicity of VU041 and VU937 in first instar *Ae. aegypti*. Values are means ± SEM based on 12 replicates of six larvae each for dH_2_O, NaCl, and KCl. Abbott’s correction [31] was applied to the percent mortalities to account for differences in the DMSO control mortality among the dH_2_O, NaCl, and KCl treatments; the mean ± SEM percent mortalities of 1% DMSO in dH_2_O, NaCl, and KCl were 7.1 ± 2.5%, 5.6 ± 3.1%, and 22.8 ± 5.3%, respectively. Lower-case letters indicate statistical categorization of the means within each small molecule treatment as determined by a one-way ANOVA with a Tukey’s multiple comparisons test (*p* < 0.05). (**B**) Effects of adding 100 mM mannitol to the rearing water on the 24 h toxicity of VU041 and VU937 in first instar *Ae. aegypti*. Values are means ± SEM based on 18 replicates of six larvae each for dH_2_O and mannitol. Abbott’s correction [31] was applied to the percent mortalities to account for differences in the DMSO control mortality between the dH_2_O and mannitol treatments; the mean ± SEM percent mortalities of 1% DMSO in dH_2_O and mannitol were 0.9 ± 0.9% and 6.5 ± 2.4%, respectively. *p* values are from unpaired *t*-tests comparing mean percent mortalities between dH_2_O and mannitol for each small molecule. The difference was considered significant if *p* < 0.05. (**C**) Relative enhancement of 24 h VU041 toxicity by mannitol, NaCl, or KCl. Values are means ± SEM based on the data in panels (A,C). Lower-case letters indicate statistical categorization of the means as determined by a one-way ANOVA with a Tukey’s multiple comparisons test (*p* < 0.05).

We next tested whether increasing the osmotic and ionic concentrations of the larval rearing water with 50 mM NaCl or KCl (100 mOsm/kg H_2_O) would exacerbate the toxic effects of VU041 or VU937 within 24 h. As shown in Figure 3a, the toxicity of VU041 in NaCl was significantly greater than that in dH_2_O and increased significantly further in KCl. On the other hand, the toxicity of VU937 in NaCl was similar to that in dH_2_O but was significantly higher in KCl, relative to that in dH_2_O and NaCl (Figure 3a). Given the significant effects of NaCl and KCl treatment on VU041’s toxicity we next tested whether 100 mM mannitol (100 mOsm/kg H_2_O) induced similar effects; mannitol was meant to increase ambient osmolality without changing NaCl or KCl concentrations. As shown in Figure 3b, the efficacy of VU041 in mannitol was significantly greater than that in dH_2_O but the efficacy of VU937 in mannitol was similar to that in dH_2_O. Figure 3c summarizes the results for VU041 in terms of the fold enhancement of toxicity (relative to that in dH_2_O). Mannitol and NaCl each enhanced the toxicity of VU041 to a similar degree (Figure 3c). On the other hand, KCl was significantly more effective than mannitol and NaCl (Figure 3c).

## 4. Discussion

The results of the present study supported our hypothesis that inhibition of Kir channels would be lethal to larval mosquitoes. The addition of either barium, a non-specific blocker of Kir channels, or VU041, a specific small-molecule inhibitor of mosquito Kir1 channels [13], to the rearing water (dH_2_O) killed first instar *Ae. aegypti* within 48 h. In contrast, the addition of VU937, a less potent small-molecule inhibitor of mosquito Kir1 channels [13], to the rearing water (dH_2_O) was less toxic than VU041. Thus, the relative toxicities of VU041 and VU937 against larvae correlated with the ability of these molecules to inhibit Kir1 channels in vitro. These results are consistent with previous toxicology studies that tested these molecules in adult female *Ae. aegypti* and *An. gambiae* [13] and adult soybean aphids, *Aphis glycines* [20]. The relatively slow time course (i.e., >24 h) for barium and VU041 to become toxic against larvae may be attributed to: (1) low permeability of the cuticle and/or gut epithelium to these molecules, thereby limiting their access to the hemolymph, as was observed previously for flonicamid [27]; and/or (2) a chronic mechanism of toxicity, such as disruption of osmoregulation.

In support of the latter notion, when we exposed larvae to an increase of ambient NaCl, KCl, or mannitol, the toxic effects of Kir inhibition by VU041 were significantly enhanced within the first 24 h. The toxic efficacy of VU041 was enhanced to a similar degree (relative to dH_2_O) by NaCl or mannitol, suggesting that Kir1 channels contribute to the acclimation of larvae to elevated ambient osmolality. The osmoregulatory acclimation may involve changes in larval drinking rates and the activities of ion transport processes in the Malpighian tubules, rectum, and anal papillae [32,33]. Remarkably, the toxic efficacy of VU041 was enhanced to a greater degree by KCl (compared to NaCl and mannitol), suggesting that Kir channels play an especially important role in the acclimation of larvae to elevated ambient K^+^ concentrations and in larval hemolymph K^+^ homeostasis. This notion is further supported by the significant enhancement of VU937’s toxic efficacy against larvae in KCl but not in NaCl or mannitol. That is, during elevated ambient KCl, even a weak inhibitor of Kir channels (VU937) caused significant larval mortality. This finding is consistent with our previous study in adult female *Ae. aegypti*, which demonstrated that Kir inhibition led to greater lethal effects when combined with challenges to hemolymph K^+^ and volume homeostasis vs. hemolymph Na^+^ and volume homeostasis [8].

One potential mechanism of toxic action by VU041 in larvae is the disruption of Malpighian tubule function. Previously we have shown that VU041 preferably inhibits mosquito Kir1 vs. Kir2B channels [13,20]. In *Ae. aegypti* larvae, Kir1 mRNA was primarily expressed in the Malpighian tubules compared to the midgut and anal papillae [21]. In adult female *Ae. aegypti*, Kir1 immunoreactivity localized to the basolateral membrane of stellate cells, where it contributed to the majority of the transepithelial K^+^ and fluid secretion [11]. Assuming a similar localization and physiological role of Kir1 in larval Malpighian tubules, VU041 treatment may impair the capacity for solute secretion in Malpighian tubules, thereby leading to a disruption of hemolymph osmotic and ionic homeostasis, especially during acclimation to elevated ambient osmolality and K^+^ concentrations. Future studies employing the Ramsay assay will be required to confirm that VU041 disrupts transepithelial ion and/or fluid secretion in larval Malpighian tubules.

We also cannot rule out that VU041 disrupts physiological processes in larval mosquitoes outside of the Malpighian tubules. For example, we have previously shown that in adult female *Ae. aegypti* the inhibition of Kir1 may disrupt the release of diuretic factors into the hemolymph that modulate the physiological responses of whole mosquito to hemolymph volume, Na^+^, and K^+^ loads [10]. Moreover, it is possible that VU041 influences drinking rates and/or ion transport in the rectum and anal papillae, which are physiological processes that contribute to larval osmoregulation [32,33]. Likewise, VU041 may impair functions of the nervous system and salivary glands, where Kir1 channels have been shown to play key physiological roles in other dipteran insects [16,17]. Additional studies will be required to fully elucidate VU041’s mechanism of toxic action in mosquito larvae and the physiological roles of Kir channels in larval osmoregulation.

## 5. Conclusions

In conclusion, our results provide the first evidence that inhibition of Kir channels in mosquito larvae induces toxicity. These findings broaden the potential applications of small molecule inhibitors of Kir channels as larvicides, pending proof that they are environmentally safe. These molecules have previously been considered tools for controlling adult female mosquitoes [8,13,19,24,25,26], but not larvae. Moreover, our findings broaden the number of small molecule inhibitors of Kir channels that should be screened for insecticidal activity. Although we have discovered hundreds of small molecule inhibitors of mosquito Kir1 channels in high-throughput screening [13,24], our insecticide discovery efforts have focused on a few small molecule inhibitors with relatively high cLog*p* values, such as VU041, for the purpose of identifying compounds that are likely to penetrate the cuticular barriers of adult female mosquitoes [13]. However, compounds with lower cLog*p* values are expected to be more water soluble, and thereby evenly disperse throughout the water column where they may be more likely to be ingested by the larvae during feeding and drinking. It remains to be determined whether Kir1 inhibitors with lower cLog*p* values possess greater larvicidal efficacy and potency than VU041.
